# Exploring the practice and attitudes of psychiatrists and psychologists towards patient-targeted googling in China

**DOI:** 10.3389/fpsyt.2024.1461514

**Published:** 2024-10-07

**Authors:** Yunzi Feng, Xinyue Hu, Yi Qiao, Yang Shao

**Affiliations:** ^1^ Shanghai Mental Health Center, Shanghai Jiao Tong University School of Medicine, Shanghai, China; ^2^ Student Counseling and Mental Health Center, Jiaxing Vocational & Technical College, Jiaxing, China

**Keywords:** patient-targeted googling (PTG), patient-therapist relationship, differences between psychiatrists and psychologists, mental health service, digital health

## Abstract

**Background:**

Patient-targeted Googling (PTG) is an unavoidable aspect of the internet era, offering both opportunities and risks. However, no PTG studies have been conducted in Asian contexts to date. Additionally, existing research has provided limited exploration of factors influencing PTG practices, particularly regarding the professional differences between psychiatrists and psychologists. This study seeks to address these research gaps.

**Method:**

A total of 943 licensed psychiatrists and psychologists working in China completed an online survey. The survey included their attitudes towards PTG (including general attitude, application situations, reasons for/against PTG) and their actual practice of PTG.

**Results:**

250(26.5%) respondents reported using PTG. Among them, 151(60.4%) respondents sought consent from clients before use, and 142(56.8%) respondents discussed search results with clients after use. Chinese psychiatrists and psychologists have contradictory attitudes, with concerns but also recognition of its possible positive effects, and expressing a need for more guidance. Compared to psychiatrists or those working in public institutions, psychologists and those working in private institutions report greater concerns about PTG but engage in it more frequently.

**Conclusions:**

Although the sample is limited, the study reveals notable differences in attitudes and practice of PTG among Chinese psychiatrists and psychologists, which may be related to their distinct professional roles and workplace environments. These findings suggest the need for further research to better understand the underlying factors contributing to these differences. Moreover, the results highlight the importance of developing tailored ethical guidelines and targeted training programs to address PTG practices for psychiatrists and psychologists in China.

## 1 Introduction

Patient Targeted Googling (PTG) refers to the practice of mental health professionals obtaining client information through online channels. This concept originated in Clinton, Silverman, and Brendel (1), where Google, the most popular search engine at that time, was used to refer to online searching behavior (2). Currently, PTG can be completed through various search engines and social media (3, 4). In fact, social media has become a part of people’s daily life, and individual information is more easily retrieved by others (5). The content published by users on these platforms, and even their browsing traces, can be obtained by others, which exposes the living habits and attitude preferences of people (6, 7).

The opportunities and risks of PTG have been extensively discussed and investigated in mental health care. On the positive side, PTG provides can enhance clinician’s understanding of patient, help identify risks and facilitate timely interventions (8–12). However, PTG also raises concerns, including potential harm to the therapeutic relationship, privacy violations, and the risk of being misled by inaccurate online information (9, 11–13). Given these complexities, it is crucial to examine the appropriateness of PTG in clinical practice and develop guidelines for its appropriate use.

Research on PTG has predominantly focused on psychologists and psychiatrists, with significant variation in reported usage rates. Among psychologists, the lowest reported rate of PTG use was 25.6% in a 2014 study from the United States (14), while the highest was 97.8% in a 2011 study conducted in the United States and Canada (14). Other studies have reported PTG rates ranging from 40% to 85% (9, 15–17). In contrast, research on PTG among psychiatrists is sparse, with one U.S. study from 2013 reporting a 35% usage rate (18). In combined samples of psychiatrists and psychologists, a 2018 study in New Zealand found that 53.4% of participants engaged in PTG (12).

On this basis, there has been relatively little research on the factors influencing PTG practices and attitudes. Among these, professional experience has been the most frequently examined, but the results remain inconsistent. Some studies have found that the more experienced psychologists are, the more lenient their attitudes toward PTG become (14, 16), though their actual practice of PTG decreases (14). In contrast, studies involving mixed samples of psychologists and psychiatrists have yielded divergent findings: a study have found that professional experience negatively predicts PTG usage (8), while the other one have found no relationship between experience, age, and PTG practices (12). Exploration of other influencing factors is almost nonexistent.

These findings provide three key insights. First, there are significant variations in the epidemiological data on PTG, suggesting that regional and temporal differences may influence PTG practices. Second, there appear to be distinctions in PTG usage between psychologists and psychiatrists. Unfortunately, no studies have directly investigated these differences, which could help us better understand the specific clinical value and risks of PTG. However, given the differences in their training and clinical approaches (19) and the data above, it is hypothesized that these two groups may have distinct perspectives on PTG. Finally, there has been limited exploration of the factors influencing PTG usage, and the results have been inconsistent while further investigation into these factors could provide better guidance for PTG practices.

In China, the rapid expansion of the internet has had a profound impact on various sectors, including mental health services. As of June 2022, there were over 1.051 billion internet users in China, with 1.027 billion social medical users (20). In such a digital milieu, it is conceivable that a considerable prevalence of PTG might be observed among psychiatrists and psychologists in China (15, 21). The use of online healthcare has seen significant growth, particularly following the Covid-19 pandemic (22, 23). In this digital environment, it is likely that PTG is becoming increasingly prevalent among Chinese psychiatrists and psychologists.

Based on the aforementioned background, this study will investigate the attitudes and practices related to PTG among Chinese psychiatrists and psychologists. The study aims to (1): provide the first set of data from Asia on PTG attitudes and practices, exploring potential cultural differences; and (2) examine the potential impact of common demographic and occupational factors on PTG attitudes and usage. A key focus of the study will be the comparison between psychiatrists and psychologists.

## 2 Materials and methods

### 2.1 Participants and procedure

The research sample for this study consisted of licensed psychiatrists, psychotherapists and psychological counselors in China. According to the Mental Health Law of China (24), psychotherapist is a qualified practitioner who can provide psychotherapy for mental disorder clients in medical institutions, while psychological counselors can only provide psychological counseling to clients in non-medical institutions such as social counseling institutions, community, schools, and other enterprises (25). Both of them can be referred to as “psychologists”, and neither of them have the right to make mental disorder diagnose or prescribe medications. Participants could have multiple identities simultaneously (like being licensed as both a psychiatrist and a psychotherapist), but the questionnaire allowed them to self-select only the one with which they most identified.

The study used an online questionnaire survey to collect data from the sample. The questionnaire was created on through an online platform powered by www.wjx.cn and shared with members of the Chinese Psychiatrist Association and the China Association for Mental Health through WeChat groups. The questionnaire was shared under the title “Online Mental Health Service Survey” to reduce respondent interest bias. The survey was conducted from April 10, 2022, to October 5, 2022. The researchers also invited members of these associations to complete the online survey and share the link with other psychologists and psychiatrists in their network. All participants were asked to answer basic demographic questions and questions related to their attitudes and practices towards PTG. A quality control question was interspersed throughout the survey to ensure data quality.

A total of 982 psychologists and psychiatrists completed the online questionnaire survey. After screening, the final analysis included 943 questionnaires, including 422 psychiatrists, 106 psychotherapists and 415 psychological counselors.

### 2.2 Questionnaire

The questionnaire consisted of three sections: personal demographic and occupational information, PTG practices, and attitudes toward PTG. The concept of PTG was explained at the outset to ensure participants had a clear understanding (1, 3).

The first section gathered demographic and occupational information, including gender, age, occupation (psychiatrist/psychotherapist/psychological counselor), highest educational level (doctor/master/undergraduate/other), years of service, primary workplace (public/private institution), and whether participants provided online mental health services.

To reduce social desirability bias, questions related to PTG practices were presented first, including whether participants had ever used PTG and whether they informed patients before or after its use.

The subsequent section focused on participants’ attitudes towards PTG, covering four key aspects: general attitudes, applicable situations, reasons for supporting PTG, and reasons against PTG. General attitudes were assessed through five items, including concerns about PTG, perceptions of PTG’s applicability in daily practice, views on the positive role of PTG in treatment or counseling, whether participants had received adequate guidance on PTG, and their desire for more explicit PTG guidelines. Responses were measured on a 7-point Likert scale (7 = fully applicable, 1 = not applicable). The section on PTG applications included seven common situations in which PTG might be used, and participants were asked to indicate their approval or disapproval of PTG in these contexts. The final section listed nine common reasons for supporting PTG and seven reasons for opposing it. Participants were asked to express their agreement or disagreement with these reasons.

**Figure 1:**
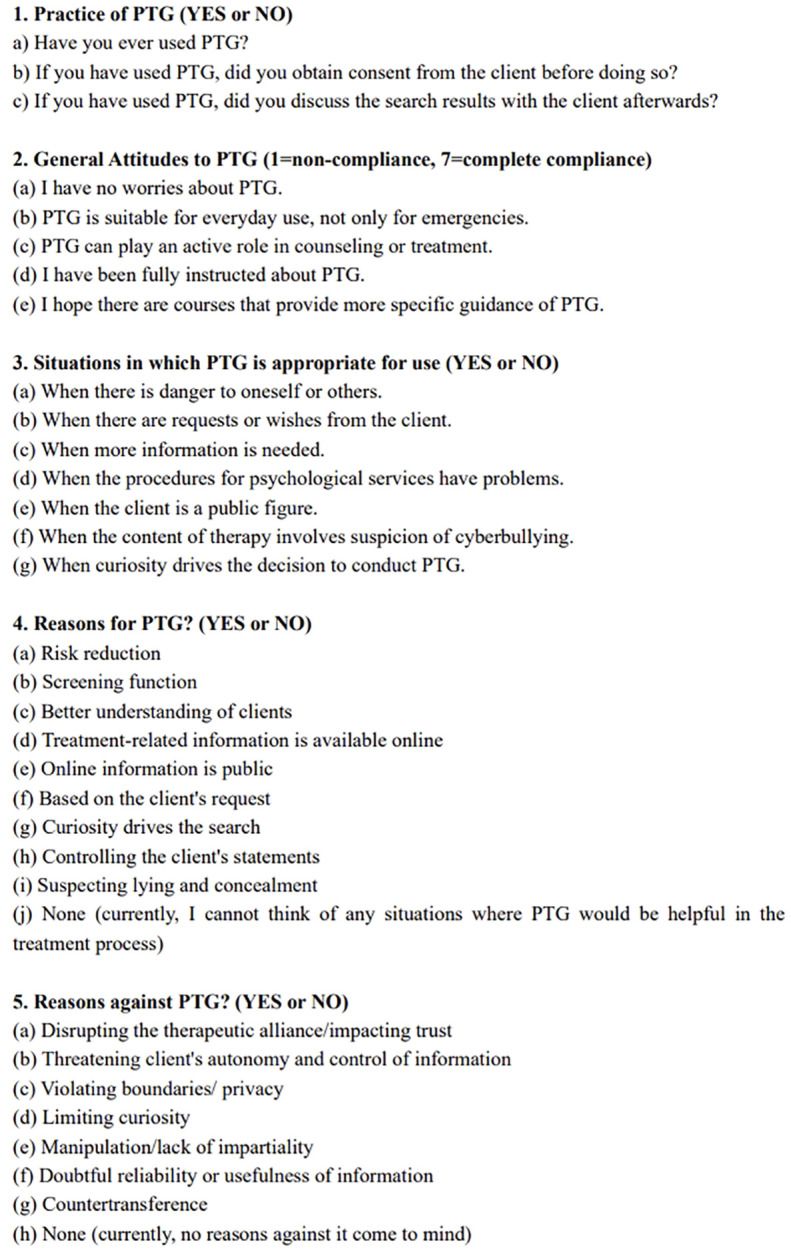
Specific items of the questionnaire.

Specific items investigating PTG attitudes and practices are detailed in **Figure 1**. All items were based on Cox’s systematic review (10) and three empirical studies were highlighted (9, 12, 26). The questionnaire was reviewed by a senior psychiatrist and a senior psychotherapist to ensure its relevance and feasibility within both professional and Chinese cultural contexts.

### 2.3 Statistical methods

The raw data was analyzed by SPSS 26.0. The frequencies and means of the demographic variables of the study were first calculated by descriptive statistics. Data were presented as means ± standard deviations.

The main statistical methods used were chi-square tests t-tests, and analysis of variance to compare the differences in the attitudes and practices of the participants in the different groups. Logistic regression analysis was used to test factors that may affect individuals’ PTG practices.

### 2.4 Ethical considerations

This study was approved by the Ethics Committee of the Shanghai Mental Health Center (IORG0002202, FWA00003065). The purpose of the study and informed consent were explained on the questionnaire homepage, and a consent form was presented before the survey began. The consent form explained that the study invited psychologists and psychiatrists to provide objective feedback on their working experience, including demographic information and attitudes towards PTG, and practices of PTG. Participants were given the choice to either agree or refuse to participate and could withdraw from the study at any time. Only participates who clicked the “agree” button could enter the formal questionnaire page to finish the survey. If participates felt uncomfortable during the survey, they could choose to end the survey and exit.

## 3 Results

### 3.1 Demographic characteristics of the participants

A total of 982 psychologists and psychiatrists completed the questionnaire, and 943 participants passed the quality control question, which consisted of 422 psychiatrists, 106 psychotherapists, and 415 psychological counselors. Since there were no significant differences found between psychotherapists and counselors in almost all items, they were grouped together under the label “psychologists” to enhance the brevity of the results without compromising accuracy. The detailed data of psychotherapists and counselors can be found in **Appendix 1** in **Supplementary Material**.

The average age of participants was 40 years, with no significant difference between psychiatrists and psychologists. The average service years for participants was 8.6 years, with psychiatrists (M = 10.58) reporting significantly longer service years than psychologists (M = 7.00). The overall sample had a higher proportion of female participants (68.7%), a characteristic that was more pronounced among psychologists (76.6%). Participants’ highest educational attainment was primarily concentrated at the undergraduate (55.8%) and master’s levels (34.7%). Among psychiatrists, a greater proportion held a bachelor’s degree (61.4%) and a doctorate (12.8%) compared to psychologists (51.2% and 3.5%, respectively), while the proportion of those holding a master’s degree was lower among psychiatrists (24.9%) than psychologists (42.6%). The majority of participants worked in public institutions (68.0%). Almost all psychiatrists were employed in public institutions (95.0%), whereas psychologists were more evenly distributed between public (46.1%) and private institutions (53.9%). Lastly, 71.3% of the overall sample reported providing online mental health services, with a lower percentage among psychiatrists (51.7%) compared to psychologists (87.1%). Detailed information is presented in **Table 1**.

**Table 1:** Basic information of the participants.

	Total	Psychiatrist	Psychologist	*χ²*	*t*
	(*N*=943)	(*N*=422)	(*N*=521)		
**Age**	40.00 ± 8.75	40.67 ± 8.58	39.45 ± 8.85		48.363
**Service Years**	8.60 ± 6.58	10.58 ± 7.50	7.00 ± 5.21		105.436**
Gender					
Male	295	173	122	33.515**	
	(31.3%)	(41.0%)	(23.4%)		
Female	648	249	399		
	(68.7%)	(59.0%)	(76.6%)		
Highest Education					
PhD	72	54	18	55.761**	
	(7.6%)	(12.8%)	(3.5%)		
Master’s Degree	327	105	222		
	(34.7%)	(24.9%)	(42.6%)		
Bachelor’s Degree	526	259	267		
	(55.8%)	(61.4%)	(51.2%)		
Other	18	4	14		
	(1.9%)	(0.9%)	(2.7%)		
Main Workplace					
Public Institution	641	401	240	256.715**	
	(68.0%)	(95.0%)	(46.1%)		
Private Institution	302	21	281		
	(32.0%)	(5.0%)	(53.9%)		
Provide online services					
Yes	672	218	454	143.326**	
	(71.3%)	(51.7%)	(87.1%)		

* p<0.05 ** p<0.01.

### 3.2 Practice of PTG

The PTG practices of participants across different groups are detailed in **Table 2**. The overall reported use of PTG in the sample was 25.6%, with a higher proportion of psychologists (34.9%) reporting PTG use compared to psychiatrists (16.1%). Additionally, younger participants (vs. older participants), those with fewer years of service (vs. those with more years of service), those working in private institutions (vs. public institutions), and those providing online services (vs. those not providing online services) reported significantly higher rates of PTG usage.

**Table 2:** Positive response rate of PTG practice in different groups (%).

	n	Q1a		n	Q1b		Q1c	
		%	χ²		%	χ²	%	χ²
**Total**	943	26.5	/	250	60.4	/	56.8	/
Job								
Psychiatrist	422	16.11	42.382**	68	57.35	0.363	60.29	0.465
Psychologist	521	34.93		182	61.54		55.49	
Gender								
Male	295	26.78	0.016	79	58.23	0.228	59.49	0.342
Female	648	26.39		171	61.40		55.56	
Age								
Below median	503	31.21	12.231**	157	58.6	0.573	55.41	0.330
Above median	440	21.14		93	63.44		59.14	
Service Year								
Below median	492	32.11	16.575**	158	60.13	0.013	55.7	0.213
Above median	451	20.4		92	60.87		58.7	
Highest Education								
PhD	72	15.28	6.197	11	18.8	9.441*	36.36	3.293
Master’s Degree	327	29.05		95	60.0		53.68	
Bachelor’s Degree	526	26.24		138	64.49		60.87	
Other	18	33.33		6	50.00		50.00	
Main Workplace								
Public Institution	641	21.06	30.518**	135	63.70	1.339	62.96	4.543*
Private Institution	302	38.08		115	56.52		49.57	
Do you provide online services?								
Yes	672	29.76	12.683**	200	59.00	0.819	55.00	
No	271	18.45		50	66.00		64.00	1.320

* p<0.05 ** p<0.01.

Among participants who had used PTG, 60.4% indicated that they sought client consent before using PTG, and 56.8% reported discussing the search results with their clients afterward. The proportion of participants seeking consent was lower among those with doctoral degrees compared to those with lower levels of education, while the proportion of participants discussing results was lower among those working in private institutions compared to those in public institutions. No statistically significant differences were found in these two items across other groups.

### 3.3 Attitudes towards PTG

**Table 3** reports participants’ general attitudes towards PTG. Overall, participants expressed concerns about the use of PTG (M = 3.109) and generally disapproved of its routine use (M = 3.716). However, their attitudes toward the positive role of PTG in treatment or counseling were neutral (M = 4.028). Participants reported receiving limited guidance on PTG (M = 2.374) but expressed a desire for more formal training or courses on the subject (M = 5.139). Comparisons between different groups revealed that psychiatrists had a more positive general attitude toward PTG compared to psychologists, men were generally more positive than women, those with postgraduate m degrees (master’s or doctorate) were more positive than those with undergraduate degrees, participants working in public institutions had a more positive attitude than those in private institutions, and those who did not provide online mental health services had a more positive attitude than those who did. Participants who practiced PTG were more likely than those who did not to believe in the positive role of PTG in treatment or counseling, perceived that they received more guidance, and had fewer concerns about PTG.

**Table 3:** General attitudes towards PTG in different groups.

	n	Q2a		Q2b		Q2c		Q2d		Q2e	
		M ± SD	t/F	M ± SD	t/F	M ± SD	t/F	M ± SD	t/F	M ± SD	t/F
**Total**	943	3.109 ± 1.682	/	3.716 ± 1.91	/	4.028 ± 1.626	/	2.374 ± 1.566	/	5.139 ± 1.762	/
Job											
Psychiatrist	422	3.20 ± 1.68	2.417	4.08 ± 1.88	27.903**	4.34 ± 1.59	28.363**	2.57 ± 1.64	12.197**	5.26 ± 1.68	3.801
Psychologist	521	3.03 ± 1.68		3.42 ± 1.88		3.78 ± 1.62		2.21 ± 1.49		5.04 ± 1.82	
Gender											
Male	295	3.33 ± 1.86	2.599**	4.09 ± 1.91	4.072**	4.40 ± 1.62	4.755**	2.76 ± 1.72	4.893**	5.29 ± 1.60	1.849
Female	648	3.01 ± 1.59		3.55 ± 1.89		3.86 ± 1.60		2.20 ± 1.46		5.07 ± 1.83	
Age											
Below median	503	3.06 ± 1.63	-0.929	3.73 ± 1.90	0.272	4.16 ± 1.59	2.622**	2.35 ± 1.54	-0.47	5.17 ± 1.72	0.597
Above median	440	3.16 ± 1.74		3.70 ± 1.92		3.88 ± 1.66		2.40 ± 1.59		5.10 ± 1.81	
Service Year											
Below median	492	3.05 ± 1.64	-1.114	3.68 ± 1.91	-0.518	4.09 ± 1.60	1.18	2.39 ± 1.54	0.242	5.23 ± 1.68	1.61
Above median	451	3.17 ± 1.72		3.75 ± 1.92		3.96 ± 1.65		2.36 ± 1.60		5.04 ± 1.84	
Highest Education											
PhD	72	2.81 ± 1.48	4.277**	3.58 ± 1.69	4.815**	3.92 ± 1.44	3.839**	2.32 ± 1.42	2.225	4.97 ± 1.76	2.498
Master’s Degree	327	2.93 ± 1.60		3.42 ± 1.90		3.80 ± 1.59		2.22 ± 1.46		4.96 ± 1.77	
Bachelor’s Degree	526	3.23 ± 1.73		3.92 ± 1.93		4.17 ± 1.64		2.46 ± 1.62		5.25 ± 1.75	
Other	18	3.89 ± 1.94		3.67 ± 1.81		4.28 ± 1.96		2.83 ± 2.12		5.61 ± 1.72	
Workplace											
Public Institution	641	3.19 ± 1.66	2.119*	3.96 ± 1.87	5.881**	4.33 ± 1.54	8.34**	2.57 ± 1.64	6.326**	5.30 ± 1.66	3.895**
Private Institution	302	2.94 ± 1.71		3.19 ± 1.90		3.39 ± 1.63		1.95 ± 1.29		4.80 ± 1.93	
Online services											
Yes	672	3.07 ± 1.68	1.087	3.56 ± 1.90	4.064**	3.85 ± 1.64	5.274**	2.35 ± 1.53	0.76	5.00 ± 1.78	3.924**
No	271	3.20 ± 1.68		4.11 ± 1.87		4.46 ± 1.50		2.44 ± 1.65		5.49 ± 1.67	
Practice of PTG											
Yes	250	3.33 ± 1.79	-2.352*	3.69 ± 1.88	0.268	4.20 ± 1.58	-2.004*	2.76 ± 1.81	-4.134**	5.16 ± 1.75	-0.262
No	693	3.03 ± 1.64		3.73 ± 1.92		3.96 ± 1.64		2.24 ± 1.44		5.13 ± 1.77	

* p<0.05 ** p<0.01.

Regarding the appropriate use of PTG, participants largely agreed that PTG is acceptable “when there is a danger to oneself or others” (83.5%), “when requested or desired by clients” (57.1%), and “when more information is needed” (50.6%). However, very few participants supported the use of PTG “when driven by mere curiosity” (8.1%). There were differences in attitudes between groups. In situations such as “when requested by clients,” “when more information is needed,” and “when there are issues with psychological services,” psychiatrists (vs. psychologists), public institution workers (vs. private institution workers), and those who do not provide online services (vs. those who provide) showed higher approval rates. Additionally, participants working in public institutions and those who do not provide online services showed higher approval rates in situations such as “when the client is a public figure” and “when the content of therapy involves suspicion of cyberbullying.” Detailed differences in participants’ attitudes toward common PTG situations are presented in **Table 4**.

**Table 4:** Positive response rate of appropriate situations of PTG in different groups (%).

	n	Q3a		Q3b		Q3c		Q3d		Q3e		Q3f		Q3g	
		%	χ²	%	χ²	%	χ²	%	χ²	%	χ²	%	χ²	%	χ²
**Total**		83.46	/	57.05	/	50.58	/	35.84	/	36.06	/	49.63	/	8.06	/
Job															
Psychiatrist	422	85.07	1.441	63.03	11.152**	59.72	25.484**	42.89	16.499**	37.91	1.146	57.11	17.096**	8.77	0.517
Psychologist	521	82.5		52.21		43.19		30.13		34.55		43.57		7.49	
Gender															
Male	295	81.02	1.851	60.00	1.523	58.31	10.24**	37.29	0.39	34.92	0.242	50.51	0.133	10.17	2.58
Female	648	84.57		55.71		47.07		35.19		36.57		49.23		7.1	
Age															
Below median	503	84.29	0.547	59.64	2.952	51.29	0.217	36.98	0.604	36.38	0.05	48.11	0.993	7.75	0.136
Above median	440	82.5		54.09		49.77		34.55		35.68		51.36		8.41	
Service Year															
Below median	492	84.35	0.594	57.93	0.321	50.41	0.013	37.2	0.818	37.8	1.366	47.56	1.76	8.54	0.316
Above median	451	82.48		56.1		50.78		34.37		34.15		51.88		7.54	
Highest Education															
PhD	72	75	4.319	55.56	6.704	50	2.263	34.72	21.753**	36.11	3.819	52.78	9.39*	2.78	6.971
Master’s Degree	327	85.02		51.68		47.4		26.3		32.11		42.81		6.12	
Bachelor’s Degree	526	83.65		60.65		52.47		42.02		38.21		53.42		9.89	
Other	18	83.33		55.56		55.56		33.33		44.44		50		11.11	
Main Workplace															
Public Institution	641	84.71	2.281	59.75	5.948*	57.41	37.321**	39.94	14.592**	38.38	4.682*	54.76	21.065**	8.89	1.874
Private Institution	302	80.79		51.32		36.09		27.15		31.13		38.74		6.29	
Do you provide online services?															
Yes	672	82.14	2.925	52.53	19.516**	44.64	33.011**	30.51	28.965**	31.7	19.269**	42.11	52.837**	6.85	4.652*
No	271	86.72		68.27		65.31		49.08		46.86		68.27		11.07	
Practice of PTG															
Yes	250	82.00	0.523	60.00	1.207	55.20	2.901	35.60	0.009	38.80	1.112	51.60	0.529	7.20	0.339
No	693	83.98		55.99		48.92		35.93		35.06		48.92		8.37	

* p<0.05 ** p<0.01.

Participants generally supported PTG for reasons such as “risk reduction” (78.6%), “screening function” (54.7%), and “better understanding of clients” (51.4%). Differences in support for PTG across various participant groups were observed. Specifically, participants who did not provide online services expressed higher support across eight of the reasons, while psychiatrists and those working in public institutions showed higher support across seven reasons. Additionally, men expressed higher support across four reasons. Differences among the other groups were less pronounced. Detailed agreement rates for supporting reasons across different groups are presented in **Table 5**.

**Table 5:** Positive response rate of reasons for PTG in different groups (%).

	n	Q4a		Q4b		Q4c		Q4d		Q4e	
		%	χ²	%	χ²	%	χ²	%	χ²	%	χ²
**Total**	943	78.58	/	54.72	/	51.43	/	39.87	/	34.89	/
Job											
Psychiatrist	422	85.07	19.125**	64.69	30.661**	61.85	33.179**	46.21	12.789**	37.91	3.079
Psychologist	521	73.32		46.64		42.99		34.74		32.44	
Gender											
Male	295	78.64	0.001	60.00	4.832*	60.00	12.618**	45.08	4.864*	36.95	0.802
Female	648	78.55		52.31		47.53		37.50		33.95	
Age											
Below median	503	79.92	1.153	55.27	0.131	54.08	3.017	40.56	0.21	37.38	2.935
Above median	440	77.05		54.09		48.41		39.09		32.05	
Service Year											
Below median	492	78.66	0.004	54.27	0.084	51.22	0.019	40.45	0.142	37.8	3.851*
Above median	451	78.49		55.21		51.66		39.25		31.71	
Highest Education											
PhD	72	75	0.724	48.61	13.828**	45.83	19.608**	37.5	8.785*	41.67	2.457
Master’s Degree	327	78.29		47.71		43.12		35.17		32.42	
Bachelor’s Degree	526	79.28		60.08		57.79		43.73		35.55	
Other	18	77.78		50.00		38.89		22.22		33.33	
Main Workplace											
Public Institution	641	83.78	32.108**	61.78	40.258**	58.81	43.673**	46.02	31.567**	38.69	12.729**
Private Institution	302	67.55		39.74		35.76		26.82		26.82	
Do you provide online services?											
Yes	672	75.74	11.165**	48.96	31.318**	45.24	35.909**	34.23	31.098**	30.36	21.138**
No	271	85.61		69.00		66.79		53.87		46.13	
Practice of PTG											
Yes	250	75.60	1.794	60.00	3.829	56.80	3.925*	42.00	0.642	39.60	3.324
No	693	79.65		52.81		49.49		39.11		33.19	
	n	Q4f		Q4g		Q4h		Q4i		Q4j	
		%	χ²	%	χ²	%	χ²	%	χ²	%	χ²
**Total**	943	45.81	/	8.8	/	21.31	/	38.18	/	14.53	/
Job											
Psychiatrist	422	49.53	4.246*	9.24	0.184	26.78	13.588**	42.65	6.489*	10.9	8.095**
Psychologist	521	42.8		8.45		16.89		34.55		17.47	
Gender											
Male	295	45.76	0	12.88	8.901**	23.73	1.491	36.61	0.446	12.54	1.363
Female	648	45.83		6.94		20.22		38.89		15.43	
Age											
Below median	503	47.91	1.917	9.34	0.395	24.25	5.554*	37.97	0.019	11.13	10.006**
Above median	440	43.41		8.18		17.95		38.41		18.41	
Service Year											
Below median	492	46.34	0.117	9.55	0.723	24.19	5.059*	38.21	0.001	13.21	1.436
Above median	451	45.23		7.98		18.18		38.14		15.96	
Highest Education											
PhD	72	41.67	1.98	6.94	8.692*	26.39	3.11	36.11	5.578	13.89	2.728
Master’s Degree	327	44.04		6.12		18.65		34.25		13.76	
Bachelor’s Degree	526	47.15		10.27		22.43		40.3		14.64	
Other	18	55.56		22.22		16.67		55.56		27.78	
Main Workplace											
Public Institution	641	47.74	2.993	9.83	2.628	24.8	14.536**	40.41	4.216*	10.3	28.864**
Private Institution	302	41.72		6.62		13.91		33.44		23.51	
Do you provide online services?											
Yes	672	41.67	16.18**	7.74	3.295	17.41	21.252**	33.78	19.148**	16.37	6.382**
No	271	56.09		11.44		31		49.08		9.96	
Practice of PTG											
Yes	250	50.40	2.886	12.40	5.487*	24.80	2.464	39.60	0.292	9.60	6.653**
No	693	44.16		7.50		20.06		37.66		16.31	

* p<0.05 ** p<0.01.

In terms of opposition to PTG, participants generally believed that PTG could “violate boundaries/privacy” (78.0%), “disrupt the therapeutic alliance” (73.4%), “threaten client autonomy and control of information” (66.1%), “lead to manipulation/lack of impartiality” (59.2%), and “limit curiosity” (51.5%). Compared to supporting reasons, there were fewer differences between participant groups regarding opposition to PTG. However, private institution workers expressed higher agreement rates across five items, and women expressed higher agreement rates across four items. Differences among the other groups were minimal. Detailed attitudes toward reasons for opposing PTG are presented in **Table 6**. It is worth adding that the results also found a significant positive correlation between the number of pro-and anti-PTG opinions identified by the participants (r=0.232, p<0.01).

**Table 6:** Positive response rate of reasons against PTG in different groups (%).

	n	Q5a		Q5b		Q5c		Q5d		Q5e		Q5f		Q5g		Q5h	
		%	χ²/F	%	χ²	%	χ²	%	χ²	%	χ²	%	χ²	%	χ²	%	χ²
**Total**	943	73.38	/	66.07	/	78.05	/	51.54	/	59.17	/	55.36	/	49.95	/	7.64	/
Job																	
Psychiatrist	422	74.17	0.243	63.27	2.663	77.73	0.047	48.1	3.605	52.84	12.665**	53.32	1.283	44.55	8.9**	8.06	0.193
Psychologist	521	72.74		68.33		78.31		54.32		64.3		57.01		54.32		7.29	
Gender																	
Male	295	68.47	5.295*	62.37	2.611	71.86	9.584**	51.19	0.021	55.25	2.729	50.51	4.081*	44.75	4.645*	7.80	0.016
Female	648	75.62		67.75		80.86		51.70		60.96		57.56		52.31		7.56	
Age																	
Below median	503	74.55	0.755	68.19	2.172	80.52	3.833	53.88	2.362	62.43	4.721*	56.26	0.359	53.08	4.237*	5.37	7.859**
Above median	440	72.05		63.64		75.23		48.86		55.45		54.32		46.36		10.23	
Service Year																	
Below median	492	74.39	0.535	66.87	0.297	80.49	3.572	50.61	0.355	61.18	1.714	56.1	0.229	52.85	3.457	6.71	1.256
Above median	451	72.28		65.19		75.39		52.55		56.98		54.55		46.78		8.65	
Highest Education																	
PhD	72	79.17	6.718	65.28	0.875	77.78	4.326	48.61	3.025	54.17	1.553	56.94	2.066	43.06	6.451	4.17	16.727**
Master’s Degree	327	77.37		67.89		80.43		54.74		60.24		55.66		55.35		4.89	
Bachelor’s Degree	526	70.15		65.21		77.19		50.38		59.51		55.51		47.72		9.13	
Other	18	72.22		61.11		61.11		38.89		50		38.89		44.44		27.78	
Main Workplace																	
Public Institution	641	72.7	0.479	63.96	3.949*	76.6	2.455	49.14	4.599*	55.69	10.026**	52.89	4.938*	44.62	22.738**	7.64	0
Private Institution	302	74.83		70.53		81.13		56.62		66.56		60.6		61.26		7.62	
Do you provide online services?																	
Yes	672	73.21	0.034	67.11	1.144	78.72	0.615	52.38	0.666	58.93	0.058	53.42	3.534	51.79	3.162	6.55	3.922*
No	271	73.8		63.47		76.38		49.45		59.78		60.15		45.39		10.33	
Practice of PTG																	
Yes	250	72.80	0.059	69.60	1.895	80.40	1.098	55.60	2.248	62.00	1.125	60.40	3.503	53.20	1.44	4.80	3.878*
No	693	73.59		64.79		77.20		50.07		58.15		53.54		48.77		8.66	

* p<0.05 ** p<0.01.

At last, a binary logistic regression analysis was conducted using participants’ demographic and attitude variables as independent variables and PTG usage as the dependent variable. The forward: LR method was used for variable selection. The final model included the following variables: psychologists (reference category: psychiatrists, B = 0.873, OR = 2.395), private institution (reference category: public institution, B = 0.762, OR = 2.143), and age (B = -0.036, OR = 0.964) among demographic factors; having received full instruction on PTG (B = 0.277, OR = 1.319) among general attitude factors; and the belief that PTG provides a better understanding of clients (B = 0.457, OR = 1.579) among supporting reasons. The Nagelkerke R² for the model was 0.158, and the results of the Hosmer-Lemeshow test indicated a chi-square value of 5.382 (p = 0.716), suggesting that the model had a good fit (**Table 7**).

**Table 7:** Binary logistic regression analysis results of PTG Practice.

	B	S.E.	Wald	p	OR	95%CI		R²
						Lower	Upper	
1 Psychologists (Reference category: psychiatrists)	0.873	0.194	20.242	0.000	2.395	1.637	3.504	0.158
2 Private Institution (Reference category: Public institution)	0.762	0.191	15.876	0.000	2.143	1.473	3.117	
3 Agreeing that PTG provide better understanding of clients(Reference category: disagreeing)	0.457	0.166	7.599	0.006	1.579	1.141	2.186	
4 I have received sufficient guidance on PTG	0.277	0.050	30.306	0.000	1.319	1.195	1.455	
5 Age	-0.036	0.009	15.356	0.000	0.964	0.947	0.982	
6 Constants	-2.972	0.524	32.214	0.000	0.051			

## 4 Discussion

### 4.1 Prevalence of PTG in China

This study presents the first set of data in the Asia (China) for PTG research. Firstly, regarding the attitudes towards PTG, the results of this survey are consistent with previous studies in Western countries (14, 26–29). Overall, Chinese psychologists and psychiatrists have concerns about PTG and hope to receive more guidance (9, 30). Although the benefits and risks of PTG vary in specific situations, PTG behavior driven by curiosity is generally resisted (9, 10). In fact, these results are also in line with the General Principles in the *American Psychological Association (APA) Ethical Principles of Psychologists and Code of Conduct* (13, 31) and previous PTG practice recommendations (32).

However, the actual use of PTG by Chinese psychiatrists and psychologists is relatively low. In this survey, only 26.5% of participants reported having used PTG, a figure significantly lower than the 53.4% reported in New Zealand in 2018 among psychiatrists and clinical psychologists (12). When examined separately, the practice rates of PTG among both psychiatrists and psychologists in this study were also lower than the usage rates reported in similar single-sample studies within the past five years (15–17, 32). Surprisingly, among those who did use PTG, the proportion of individuals who obtained client consent before conducting PTG (60.38%) and the proportion who discussed the search results with the client afterward (56.98%) were much higher than the 16% to 40% reported in the systematic review by Cox (10).

We suggest that the lower prevalence of PTG in China may be associated with the cautious attitude of Chinese people towards self-disclosure on social networking sites (33). Previous research has consistently found that East Asians, including Chinese individuals, tend to express less self-sensitive information than Westerners (34, 35). Despite some concerns about privacy, Americans are generally more comfortable with sharing personal information online, as they perceive more benefits and lower risks from social network activities and have greater trust in service providers and legal protections (36, 37). Chinese people tend to be more reserved in their self-expression on social networking sites, and this attitude may also affect their perception of online information quality and reliability, leading to a lower likelihood of PTG. Additionally, China has a lower number of mental health practitioners per capita than the United States, which could result in more workload and pressure on Chinese psychologists and psychiatrists (38). As a result, they may prefer to use effective and rapid information collection methods, such as direct questioning of parents (39), rather than PTG.

### 4.2 Differences between attitudes and practices

This study revealed three notable and significant patterns: psychologists (vs. psychiatrists), those working in private institutions (vs. public institutions), and individuals providing online services (vs. not providing online services) expressed greater concerns about PTG but also demonstrated a higher reliance on its use in practice.

For psychologists, this may be linked to their emphasis on the therapeutic alliance. “Disrupting the therapeutic alliance/impacting trust” is one of the primary reasons many professionals oppose PTG. The therapeutic alliance is a critical element of psychotherapy (40, 41), often directly influencing the success or failure of treatment (42, 43). In contrast, psychiatrists primarily rely on medication as a treatment modality, and the therapeutic alliance is relatively less significant for medication efficacy (44, 45). This distinction makes psychologists more cautious about the potential negative impacts of PTG. Additionally, psychologists are more involved in processes such as transference and empathy, making them more sensitive to ethical concerns like privacy and justice (46, 47).

But surprisingly, despite the presence of more concerns, psychologists gave more practice to PTG. This may be due to differences in their working resources. Psychiatrists often rely on a variety of objective sources, such as blood tests, symptom scales, family observations and so on, to formulate diagnoses and treatment plans with a well-rounded view. In this case, the benefits of PTG practice may not seem necessary. In contrast, psychologists depend heavily on the subjective information provided by clients during therapy sessions (48, 49). It is challenging for novice therapists to navigate, extract, and make sense of the information reported by patients, prompting them to seek additional data through PTG. Moreover, research indicates that few participants have fully considered the potential risks of PTG before engaging in it (50), suggesting that situational factors play a significant role in this behavior. Psychologists, in particular, tend to spend more time with individual cases—for example, seeing 2 to 3 clients in a morning session, compared to psychiatrists who may see over 10 patients in the same timeframe (19). This gives psychologists more opportunities to conduct PTG searches. Additionally, the covert nature of PTG, being largely anonymous and private, may reduce perceived risks, leading psychologists to engage in PTG despite their concerns. A large-scale study conducted in the United States on psychology doctoral students found that two-thirds of participants, despite disapproving of PTG, had used it at least once. The study attributed this to the frequent use of the internet by graduate students (14).

A similar pattern was observed among participants working in private institutions. They expressed more negative attitudes towards PTG compared to their counterparts in public institutions, reported higher usage rates. One of the important reasons may be that psychologists are the main service members in private institutions, and there are few psychiatrists. However, after the occupational difference is included in the regression equation, the prediction function of private institutions is still significant, which shows that its unique aspects have not been explained. The characteristics of clients in private institutions may be a contributing factor. Private institutions generally have fewer clients than public institutions (25, 51), and their clients often have higher expectations for service quality (52). A studies have found that high patient concerns about privacy predict negative perceptions of PTG (53), and privacy issues are important reasons for many clients to choose more expensive private institutions. This drives professionals to be more cautious in their practice to avoid negative experiences that could lead to client loss. However, the covert nature of PTG may allow private institution staff to engage in it despite their concerns. Additionally, many private institutions that emerged during the current mental health boom are registered with local business bureaus rather than health authorities, and a significant number of their staff may lack formal degrees in psychiatry or psychology (25, 54), resulting in weaker awareness of ethical issues. The focus on attracting clients in private institutions, where social media and search engine advertising are common strategies (30), may also make PTG use more instinctive.

Finally, the differences in attitudes and practices among those providing online services may be due to the overlap with the characteristics of psychologists and those working in private institutions. The logistic regression analysis showed that psychologists (OR=4.380, p<0.01) and private institution staff (OR=4.740, p<0.01) were the strongest predictors for providing online services. And in the regression model predicting PTG usage, “providing online services” was not included in the equation, yet psychologists and private institution staff still demonstrated significant predictive power.

### 4.3 Predictors of PTG practice

The logistic regression results showed that professional background and workplace were the strongest predictors of PTG use among the demographic variables. This was followed by whether participants had received sufficient PTG guidance (as part of general attitudes) and the belief that PTG could help in better understanding clients (as part of supporting attitudes). Although age had a statistically significant effect, its influence was relatively weak. Gender, service years and whether online service did not affect PTG usage.

The impact of professional background and workplace on PTG practice has been discussed in detail in previous sections. Regarding attitudes, it is not surprising that recognizing PTG as a way to obtain more comprehensive information, which can promote understanding and empathy towards clients, serves as a predictor of PTG use. The core purpose of PTG is to gather more information about clients (55). If practitioners believe that this additional information will benefit their clients, they are more likely to engage in PTG. Furthermore, adequate guidance can help individuals better understand the concept and methods of PTG, increasing their confidence in using it (56).

However, it is essential to consider whether the additional information provided by PTG truly contributes to a better understanding of the client, or if it merely reflects a reliance on external information due to a lack of experience. PTG can be seen as a shift from “analysis” to “action (17),” which makes the problem less challenging but also hinders the development of the therapist’s professional competence. From another perspective, search may also be evidence that the therapeutic alliance is not yet stable (57). In fact, some studies have found correlations between more experience and less search frequency (8, 14). For senior psychodynamic therapists, the fantasies described by patients is even more valuable than the actual reality (17, 50). Therefore, both psychiatrists and psychotherapists should be cautious about relying on PTG, as overreliance may hinder the development of professional skills.

In addition, the study found that age had a significant, albeit weak, effect on PTG use, with younger professionals being more likely to engage in PTG. This may be because younger individuals, as digital natives, are more familiar with and reliant on the internet (30). However, the average age of participants in this study was 40, which is not particularly old, and many professionals have started providing online treatment during the pandemic, making them also familiar with internet usage. Therefore, the influence of age on PTG practice is not as pronounced.

Last but not least, professional experience is often thought to influence practitioners’ PTG practices. However, in both the group comparisons of attitude items and the final regression model of PTG practice, the impact of professional experience was not confirmed. While the use of years of service as a proxy for professional experience in this study may not have been entirely accurate, a similar study conducted with a mixed sample of psychiatrists and psychotherapists in New Zealand reported comparable results (12). Therefore, further validation of this relationship is needed in future research.

### 4.4 Contradiction between support and opposition

Beyond the differences in attitudes and practices, the study also revealed a positive correlation between the number of reasons participants endorsed for supporting PTG and those for opposing it. This suggests an internal conflict among participants regarding PTG, which is understandable given the clear potential risks and benefits of PTG. Existing literature has extensively discussed the potential risks and benefits of PTG, such as whether PTG violates client privacy, disrupts the therapeutic relationship, or impacts treatment outcomes (10, 17, 29). On the other hand, completely rejecting PTG might result in missed opportunities for deeper understanding and timely intervention for patients (3, 11). With the increasing prevalence of digitalization and the refinement of social media algorithms, psychiatrists and Psychologists may face more situations requiring PTG decisions, sometimes even involuntarily (e.g., client profiles being suggested on social media platforms).

Unfortunately, the study indicates that psychiatrists and psychologists have received very limited guidance on PTG (58), despite expressing a strong need for it. Therefore, providing clear guidelines and instructional materials on whether and how to use PTG is crucial. Cole (59), in a review of PTG literature, recommended that decisions about PTG should always be based on the patient’s specific circumstances and should prioritize the “best interests of the patient.” The risk-benefit framework can be used to assess the necessity and impact of PTG, guiding professionals in conducting ethically permissible searches while considering the intrusiveness of PTG and its potential impact on the therapeutic relationship (1, 59).

If PTG is deemed necessary, obtaining the client’s consent in advance may be a better approach. This would not only ensure the accuracy of the information gathered but might also strengthen the therapeutic alliance by demonstrating the therapist’s concern for the patient (50). If the client refuses, the professional can gain further insight into the patient through the act of refusal and discussions around it.

### 4.5 Limitations

Despite efforts to refine it, this study still has some limitations. Due to the limitation of objective factors, the study did not conduct focus group discussions during the design of the questionnaire, which may lead to limitations in the applicability and diversity of the questionnaire. In addition, the collection of professional experience in the questionnaire depends on the years of practice reported by the participants, but the years and experience may not be exactly matched. For example, a psychologist with ten years of experience and five cases per week may not be that senior. In addition, the questionnaire was forwarded through WeChat groups, and it may have a low response rate. At the same time, participants’ reports of PTG may also be affected by social expectation bias. Additionally, this study only included psychiatrists and psychologists, and the attitudes and practices of other mental health professionals are worth exploring in the future.

## 5 Conclusion

Although the sample size is limited, this study reveals significant differences in attitudes toward PTG among Chinese psychiatrists and psychologists, likely influenced by their distinct professional roles and workplace environments. Our findings suggest that psychologists and practitioners in private institutions engage in PTG more frequently, while simultaneously harboring greater concerns about its use. This contradiction reflects their reliance on the therapeutic alliance and the high expectations of clients in private institutions.

These results highlight the unique challenges and potential risks associated with PTG practices in Chinese psychiatrists and psychologists. Future research should further explore the underlying factors contributing to these differences to better understand the role of PTG in different professional contexts. Moreover, the findings underscore the urgent need to develop ethical guidelines tailored to the Chinese context and to provide targeted training for both psychiatrists and psychologists to ensure that PTG practices are conducted safely and responsibly for both clients and professionals.

## Data Availability

The raw data supporting the conclusions of this article will be made available by the authors, without undue reservation.
