# Supplementary material for: Exploring the practice and attitudes of psychiatrists and psychologists towards patient-targeted googling in China
Source: Front Psychiatry. 2024 Oct 7;15:1461514. doi: 10.3389/fpsyt.2024.1461514 (PMC11491373; doi:10.3389/fpsyt.2024.1461514)
Supplement: Supplementary file 1 [file Table1.docx]

**Appendix 1**

**Comparison between psychotherapists and psychological consultants**

These tables represent the section where the items with significant differences were compared pairwise after conducting chi-square tests among the three groups of psychiatrists, psychotherapists, and counselors. From there tables below we can see that except for the statistical information, there were no significant differences between the attitudes and practices of psychological counselors and psychotherapists in all survey items.

**Table 1-1a. Chi-square Test of Demographics**

| Items | Answer | Profession(%) | | Total | χ2 | *p* |
| --- | --- | --- | --- | --- | --- | --- |
|  |  | Therapist | Consultant |  |  |  |
| Gender | Male | 35(33.02) | 87(20.96) | 122(23.42) | 6.842 | 0.009** |
|  | Female | 71(66.98) | 328(79.04) | 399(76.58) |  |  |
| Highest Education | PhD | 1(0.94) | 17(4.10) | 18(3.45) | 5.433 | 0.143 |
|  | MD | 39(36.79) | 183(44.10) | 222(42.61) |  |  |
|  | BD | 62(58.49) | 205(49.40) | 267(51.25) |  |  |
|  | Others | 4(3.77) | 10(2.41) | 14(2.69) |  |  |
| Provide online services? | No | 31(29.25) | 36(8.67) | 67(12.86) | 31.883 | 0.000** |
|  | Yes | 75(70.75) | 379(91.33) | 454(87.14) |  |  |
| Main Workplace | Public Institution | 94(88.68) | 146(35.18) | 240(46.07) | 97.265 | 0.000** |
|  | Private Institution | 12(11.32) | 269(64.82) | 281(53.93) |  |  |
| Practice of PTG? | No | 76(71.70) | 263(63.37) | 339(65.07) | 2.574 | 0.109 |
|  | Yes | 30(28.30) | 152(36.63) | 182(34.93) |  |  |
| * *p*<0.05 ** *p*<0.01 | | | | | | |

**Table 1-1b. Post hoc LSD comparison of ANOVA of demographic variables**

|  | Profession1 | Profession2 | *M1* | *M2* | *M1-M2* | *p* |
| --- | --- | --- | --- | --- | --- | --- |
| Age | Therapist | Consultant | 36.774 | 40.14 | -3.366 | 0.000** |
| Service Time | Therapist | Consultant | 6.726 | 7.075 | -0.348 | 0.614 |

* *p*<0.05 ** *p*<0.01

**Table 1-2a. Chi-square Test of general attitude**

|  | Profession1 | Profession2 | *M1* | *M2* | *M1-M2* | *p* |
| --- | --- | --- | --- | --- | --- | --- |
| 2 PTG is suitable for everyday use, not only for emergencies. | Therapist | Consultant | 3.462 | 3.414 | 0.048 | 0.816 |
| 3 PTG can play an active role in counseling or treatment. | Therapist | Consultant | 3.972 | 3.728 | 0.244 | 0.162 |
| 4 I have been fully instructed about PTG. | Therapist | Consultant | 2.368 | 2.176 | 0.192 | 0.257 |
| * *p*<0.05 ** *p*<0.01 |  |  |  |  |  |  |

**Table 1-3a. Chi-square Test of Appropriate Use of PTG**

| Items | Answer | Profession(%) | | Total | χ2 | *p* |
| --- | --- | --- | --- | --- | --- | --- |
|  |  | Therapist | Consultant |  |  |  |
| 1 When there is danger to oneself or others. | No | 17(16.04) | 76(18.31) | 93(17.85) | 0.298 | 0.585 |
|  | Yes | 89(83.96) | 339(81.69) | 428(82.15) |  |  |
| 2 When there are requests or wishes from the client. | No | 54(50.94) | 195(46.99) | 249(47.79) | 0.529 | 0.467 |
|  | Yes | 52(49.06) | 220(53.01) | 272(52.21) |  |  |
| 3 When more information is needed. | No | 51(48.11) | 245(59.04) | 296(56.81) | 4.106 | 0.043* |
|  | Yes | 55(51.89) | 170(40.96) | 225(43.19) |  |  |
| 4 When the procedures for psychological services have problems. | No | 64(60.38) | 300(72.29) | 364(69.87) | 5.69 | 0.017* |
|  | Yes | 42(39.62) | 115(27.71) | 157(30.13) |  |  |
| 5 When the client is a public figure. | No | 77(72.64) | 264(63.61) | 341(65.45) | 3.043 | 0.081 |
|  | Yes | 29(27.36) | 151(36.39) | 180(34.55) |  |  |
| 6 When the content of therapy involves suspicion of cyberbullying. | No | 62(58.49) | 232(55.90) | 294(56.43) | 0.23 | 0.632 |
|  | Yes | 44(41.51) | 183(44.10) | 227(43.57) |  |  |
| 7 When curiosity drives the decision to conduct PTG. | No | 99(93.40) | 383(92.29) | 482(92.51) | 0.149 | 0.699 |
|  | Yes | 7(6.60) | 32(7.71) | 39(7.49) |  |  |

* *p*<0.05 ** *p*<0.01

**After Bonferroni correction (α=0.0167), there was no difference between the two groups.**

**Table 1-4a. Chi-square Test of Reasons for PTG**

| Items | Answer | Profession(%) | | Total | χ2 | *p* |
| --- | --- | --- | --- | --- | --- | --- |
|  |  | Therapist | Consultant |  |  |  |
| 1 Risk reduction | No | 22(20.75) | 117(28.19) | 139(26.68) | 2.388 | 0.122 |
|  | Yes | 84(79.25) | 298(71.81) | 382(73.32) |  |  |
| 2 Screening function | No | 57(53.77) | 221(53.25) | 278(53.36) | 0.009 | 0.924 |
|  | Yes | 49(46.23) | 194(46.75) | 243(46.64) |  |  |
| 3 Better understanding of clients | No | 55(51.89) | 242(58.31) | 297(57.01) | 1.423 | 0.233 |
|  | Yes | 51(48.11) | 173(41.69) | 224(42.99) |  |  |
| 4 Treatment-related information is available online | No | 68(64.15) | 272(65.54) | 340(65.26) | 0.072 | 0.788 |
|  | Yes | 38(35.85) | 143(34.46) | 181(34.74) |  |  |
| 5 Online information is public | No | 76(71.70) | 276(66.51) | 352(67.56) | 1.039 | 0.308 |
|  | Yes | 30(28.30) | 139(33.49) | 169(32.44) |  |  |
| 6 Based on the client's request | No | 62(58.49) | 236(56.87) | 298(57.20) | 0.091 | 0.763 |
|  | Yes | 44(41.51) | 179(43.13) | 223(42.80) |  |  |
| 7 Curiosity drives the search | No | 95(89.62) | 382(92.05) | 477(91.55) | 0.642 | 0.423 |
|  | Yes | 11(10.38) | 33(7.95) | 44(8.45) |  |  |
| 8 Controlling the client's statements | No | 88(83.02) | 345(83.13) | 433(83.11) | 0.001 | 0.978 |
|  | Yes | 18(16.98) | 70(16.87) | 88(16.89) |  |  |
| 9 Suspecting lying and concealment | No | 62(58.49) | 279(67.23) | 341(65.45) | 2.851 | 0.091 |
|  | Yes | 44(41.51) | 136(32.77) | 180(34.55) |  |  |
| 10 None (currently, I cannot think of any situations where PTG would be helpful in the treatment process) | No | 90(84.91) | 340(81.93) | 430(82.53) | 0.519 | 0.471 |
|  | Yes | 16(15.09) | 75(18.07) | 91(17.47) |  |  |
| * *p*<0.05 ** *p*<0.01 | | | | | | |

**Table 1-5a. Chi-square Test of Reasons against PTG**

| Items | Answer | Profession(%) | | Total | χ2 | *p* |
| --- | --- | --- | --- | --- | --- | --- |
|  |  | Therapist | Consultant |  |  |  |
| 1 Disrupting the therapeutic alliance/impacting trust | No | 30(28.30) | 112(26.99) | 142(27.26) | 0.074 | 0.786 |
|  | Yes | 76(71.70) | 303(73.01) | 379(72.74) |  |  |
| 2 Threatening client's autonomy and control of information | No | 31(29.25) | 134(32.29) | 165(31.67) | 0.362 | 0.548 |
|  | Yes | 75(70.75) | 281(67.71) | 356(68.33) |  |  |
| 3 Violating boundaries/ privacy | No | 27(25.47) | 86(20.72) | 113(21.69) | 1.121 | 0.29 |
|  | Yes | 79(74.53) | 329(79.28) | 408(78.31) |  |  |
| 4 Limiting curiosity | No | 48(45.28) | 190(45.78) | 238(45.68) | 0.009 | 0.926 |
|  | Yes | 58(54.72) | 225(54.22) | 283(54.32) |  |  |
| 5 Manipulation/lack of impartiality | No | 40(37.74) | 146(35.18) | 186(35.70) | 0.24 | 0.624 |
|  | Yes | 66(62.26) | 269(64.82) | 335(64.30) |  |  |
| 6 Doubtful reliability or usefulness of information | No | 47(44.34) | 177(42.65) | 224(42.99) | 0.098 | 0.754 |
|  | Yes | 59(55.66) | 238(57.35) | 297(57.01) |  |  |
| 7 Countertransference | No | 54(50.94) | 184(44.34) | 238(45.68) | 1.485 | 0.223 |
|  | Yes | 52(49.06) | 231(55.66) | 283(54.32) |  |  |
| 8 None (currently, no reasons against it come to mind) | No | 95(89.62) | 388(93.49) | 483(92.71) | 1.871 | 0.171 |
|  | Yes | 11(10.38) | 27(6.51) | 38(7.29) |  |  |
| * *p*<0.05 ** *p*<0.01 | | | | | | |
